# Pulmonary Artery Aneurysm in Behcet Disease: Medical, Endovascular or Surgical Intervention

**DOI:** 10.7759/cureus.49368

**Published:** 2023-11-24

**Authors:** Isha Samreen, Puja Darji, Satchel Genobaga, Saivishnu Doosetty, Tamanna Mohta, Gargi Maity, Chong Vue, Sriharsha Nakka, Chukwuemeka Umeh

**Affiliations:** 1 Internal Medicine, Hemet Global Medical Center, Hemet, USA; 2 Infectious Disease, Hemet Global Medical Center, Hemet, USA

**Keywords:** review article, hemoptysis, behcet’s syndrome, vascular complications, endovascular interventions, pulmonary artery aneurysm

## Abstract

Behçet's disease is a chronic inflammatory condition that predominantly affects the body's blood vessels, exhibiting various clinical manifestations and complications. The exact cause remains unclear, but genetic predisposition, immune responses, and vascular activation are believed to contribute to its development. This disease is more prevalent in certain geographic regions and primarily affects young adults, particularly males. Pulmonary aneurysm, a complication of Behçet's disease, is the leading cause of mortality in Behcet disease. In this review, we summarize the complications of Behcet disease with a focus on pulmonary artery aneurysms. We discussed the medical, endovascular, and surgical management of pulmonary aneurysms in Behcet disease and the indications and outcomes of the different treatment options. Corticosteroids and cyclophosphamide remain the preferred first-line therapy. However, clinical improvement with infliximab or adalimumab, tumor necrosis factor-alpha (TNFα) blocking agents, have been reported after treatment failure with recommended first-line agents. In patients who fail medical therapy or those with life-threatening hemoptysis, endovascular or surgical intervention is the next option. Endovascular interventions include pulmonary artery embolization with coils or acrylic glue and using plugs, occluders, or stents. Endovascular interventions usually have fewer adverse effects than surgery. Although the risk of surgical procedures is high in pulmonary artery aneurysms, it could be a life-saving procedure in patients with life-threatening hemoptysis. Surgical options, including pulmonary artery ligation, aneurysmorrhaphy, segmentectomy, lobectomy, or pneumonectomy are available. However, the results of surgical therapy for Behçet aneurysms are often disappointing.

## Introduction and background

Behçet's disease, or Behçet's syndrome, is a chronic inflammatory condition primarily affecting the body's blood vessels. Common symptoms include recurring painful oral and genital ulcers, inflammation of the eyes (uveitis), and skin lesions. It can also impact other areas, such as the gastrointestinal tract, joints, nervous system, and blood vessels, leading to complications [1]. This disease is more prevalent in regions like the Mediterranean, Middle East, and Asia, often affecting young adults, with males experiencing more severe manifestations. It is a rare disease in the United States, with an estimated prevalence of 0.33 to 5.2 people per 100,000 population [2].

The exact cause of Behçet's syndrome remains unclear, but it likely involves abnormal immune responses triggered by specific exposures in individuals with a genetic predisposition. The disease exhibits characteristics of both autoimmunity and autoinflammation. Genetic influences, altered immune responses, vascular activation causing clotting, and epigenetic modifications contribute to its development. The genetic predisposition for Behçet's syndrome is likely polygenic, involving multiple genes. Notably, human leukocyte antigens (HLA), especially HLA-B51, significantly increase the risk of developing the disease. Besides HLA genes, various non-HLA genes related to immune response, endothelial function, and cytokines also influence disease susceptibility and severity. Certain genetic variations are associated with the onset and severity of disease. It's evident that genetic factors, both HLA and non-HLA genes, play a pivotal role in the susceptibility to and severity of Behçet's syndrome [3-5]. Other factors contributing to the disease include molecular mimicry, where bacterial antigens might cross-react with human peptides, especially heat shock proteins, potentially triggering the disease. Specific bacteria, like streptococci, are thought to affect immune responses in individuals with Behçet's syndrome. There are also notable alterations in the immune system, including changes in T cell populations, cytokine production, and the formation of autoantibodies, all of which are key factors contributing to the disease. Additionally, the inflammatory response and vascular damage seen in Behçet's syndrome involve endothelial activation, coagulation abnormalities, and neutrophil activation. These factors collectively contribute to the complex nature of Behçet's syndrome [3,5].

Epidemiology of pulmonary aneurysm in Behcet disease 

Behcet's disease is commonly found in populations originating along the 'Silk Road." This term refers to a trade route that extended from China to Mediterranean nations [6]. This association commonly includes the Mediterranean, Turkey, Japan, and Iran. Among these, the prevalence is highest in Turkey, with 80-370 cases per 100,000 [7]. Furthermore, the age group widely associated with the disease is those between 20 and 40. Studies have found that the gene marker HLA-B51 may be a risk factor for developing Behcet's disease, as found in 60% of those with Behcet's disease. Amongst risk factors, environmental contributions cannot be excluded [8].

Behcet's disease can include the vascular system 25-30% of the time [9]. Pulmonary involvement in Behcet's disease is not a common occurrence, as overall incidence is reported at less than 5%, of which pulmonary arterial involvement is most common. Pulmonary artery aneurysms tend to be the most life-threatening complication of Behcet syndrome [10].

## Review

Clinical manifestations and complications of Behcet disease 

Behcet's syndrome is a multisystem inflammatory disorder with many clinical manifestations and complications, generally secondary to vascular inflammation in small and large vessels [11]. The most prominent feature is painful mucocutaneous ulcers. Oral ulcers are similar to aphthous ulcers, with well-defined borders, necrotic bases, and surrounding erythema. Ulcers are exclusively internal and do not involve the outer portion of the lip [12,13]. Genital ulcers are also painful and have features similar to oral ulcers. Skin involvement varies from pustules to pyoderma gangrenosum and erythema multiforme.

Common ocular manifestations include uveitis, conjunctivitis, and hypopyon [14-16]. The course of the ocular involvement is typically recurrent, unilateral, and bilateral exacerbations, followed by spontaneous resolution. Ocular involvement is characterized by inflammation and neovascularisation of the different visual layers [17]. The uveitis can lead to ocular complications such as macular edema, posterior synechiae, cataracts, optic atrophy, and glaucoma, which can cause permanent vision loss [18,19].

Neurological manifestations occur in about 9% of Behcet's disease patients [20]. Neurological involvement can be divided into parenchymal and non-parenchymal (vessel diseases such as vasculitis and thrombosis) [21-23]. More commonly, direct parenchymal inflammation occurs. This manifests as a brainstem syndrome manifesting as ophthalmoparesis and ataxia with a subacute progression [24]. Less commonly, venous sinus or superficial cerebral venous thrombosis can occur, presenting with headache and visual changes. Cognitive dysfunction, psychiatric disorders, and peripheral nervous system complications can also occur, although more rarely [25].

Gastrointestinal complications can also occur due to diseased small or large vessels, leading to ulcers, ischemia, and thrombosis. Ulcers are most commonly located in the ileocecal region, caused by inflammation around the vasa vasorum and the resulting medial destruction [26]. Large vessel thrombosis can occur in the hepatic veins, leading to Budd-Chiari syndrome.

Vascular complications occur in up to half of patients with Behcet's syndrome, and venous manifestations are more common than arterial. Venous manifestations include superficial and deep venous thrombosis and thromboses of the hepatic veins, superior vena cava, and inferior vena cava [27]. Thrombogenesis in Behcet's is mediated by inflammation rather than traditional cardiovascular risk factors. In this disease process, endothelial dysfunction and platelet activation are a product of neutrophil-mediated mechanisms [4].

Arterial manifestations include thrombosis and aneurysm [28]. One prominent location of aneurysm is the pulmonary artery. Pulmonary artery aneurysms mainly involve large and proximal pulmonary artery branches [29-31] and are a rare complication in Behcet's syndrome, with around 1% prevalence [32]. Pulmonary vasculitic aneurysms are usually only seen in Behcet syndrome and Hughes-Stovin syndrome, a variant of Behcet disease [29-31,33].

Pulmonary aneurysm in Behcet Disease

Pulmonary artery aneurysms are Behcet disease's most common pulmonary manifestation, and hemoptysis is the most common clinical manifestation of pulmonary aneurysms. Pulmonary aneurysms can occur as single or multiple, unilateral or bilateral, with multiple bilateral aneurysms being more common [34,35]. The pathogenesis of the pulmonary artery aneurysm is likely due to obliterative endarteritis of the vasa vasorum, resulting in aneurysm formation or perforations and pseudoaneurysm formation [36]. The aneurysms vary in size, and sizes ranging from 1 to 7 centimeters were reported by Tunaci et al., 1999 in their analysis of 46 pulmonary aneurysms [35]. They are more common in males and usually co-exist with other extrapulmonary vessel lesions, such as extrapulmonary arterial aneurysms and deep venous thrombosis [31,34,37]. Partial or complete thrombosis within the aneurysm occurs in about a third of pulmonary aneurysms [35]. Pulmonary aneurysms can rupture and bleed into the pulmonary tissue and heal with fibrosis. They could also rupture into the bronchial lumen and cause hemoptysis, Behcet disease's leading cause of death [31,35]. A pulmonary angiogram is the gold standard for diagnosing pulmonary aneurysms. However, it may be risky in Behcet disease patients as the venous puncture or rapid injection of contrast may initiate or worsen an existing thrombus [35]. Additionally, a pulmonary angiogram may not detect thrombosed pulmonary aneurysms. Thus, helical computed tomography angiography (CTA) has emerged as a non-invasive and excellent way of detecting pulmonary aneurysms in Behcet disease [36,38]. Magnetic resonance imaging (MRI) could be an alternative option for patients who cannot get a CTA, such as those with impaired renal function or severe iodine allergy [39,40].

Treatment

Different treatment modalities have been used to manage pulmonary artery aneurysms, including immunosuppression, anticoagulation, surgery, and embolization. There have been no randomized controlled trials to evaluate these treatment options, and the recommendations and guidelines are based on nonrandomized trials, observational studies, and expert opinions [34].

Medical management

The primary goal in medically managing pulmonary aneurysms in Behcet disease is to suppress the inflammatory response to avoid irreversible end-organ damage. The European League Against Rheumatism (EULAR) guidelines for managing Behcet disease pulmonary and peripheral artery aneurysms recommend glucocorticoid and cyclophosphamide as first-line treatment [41]. Previously published case reports have also provided evidence of improved pulmonary disease in Behcet disease using immunosuppressants like cyclophosphamide, azathioprine, cyclosporine, mycophenolate, colchicine, and glucocorticoids. One such case series of 26 patients by Hamuryudan et al. reported that intravenous pulses of corticosteroids and cyclophosphamide followed by long-term oral corticosteroids with oral cyclophosphamide or azathioprine improved 5-year survival, from approximately 40% to 80% [29]. In these patients, prednisolone was started at 1 mg/kg/day, tapered for 2 to 5 months, and discontinued if possible. Additionally, the patients received intravenous pulses of cyclophosphamide (1 g monthly during the first year and every two months after that) for two years. After two years, cyclophosphamide was either continued or replaced with azathioprine (2.5 mg/kg/d). Following these findings, the EULAR recommends the treatment of pulmonary aneurysms with cyclophosphamide for at least two years, followed by azathioprine [41].

Furthermore, there have been multiple reports of clinical improvement with infliximab or adalimumab, tumor necrosis factor-alpha (TNFα) blocking agents, after treatment failure with recommended first-line agents [42-47] or as part of first-line treatment in patients with life-threatening pulmonary aneurysms [48]. Anti-TNFα agents are in the EULAR recommendations for managing aspects of Behcet syndrome, such as refractory eye, skin, mucosal, or neurological disease; however, they are not recommended for major vessel disease [41]. They have, however, demonstrated benefit in cases refractory to cyclophosphamide or azathioprine [42]. In a multicenter study of 18 refractory Behcet disease patients with major vessel involvement, including four with pulmonary aneurysms, the 12-month risk of relapse was significantly lower with anti-TNFα compared with conventional immunosuppressants (i.e., cyclophosphamide, azathioprine or methotrexate) in previous treatment lines 6% vs. 41% (p = 0.036) [49].

Medical treatment alone results in the resolution of the aneurysm in many patients. Tunaci et al. 1999 did a computed tomography (CT) follow-up of 46 Behcet patients with pulmonary aneurysms who were medically treated. They found that 76% of the aneurysms had completely resolved within 3-42 months (mean of 21 months) after treatment, and the remaining 24% had regressed in size. They discovered that the process of aneurysm resolution with medical treatment involves thrombosis in the aneurysm, then lysis of the thrombosis, and eventual regression or disappearance of the aneurysm [35].

Despite the efficacy of medical treatment, the size of the aneurysm is associated with prognosis and mortality. Seyahi et al. found that patients with pulmonary aneurysms diameter ≥3 cm were more likely to die from complications compared to those with a diameter <3 cm (p = 0.002) [30].

Endovascular management of pulmonary aneurysm in Behcet disease 

Endovascular management becomes an option in patients with pulmonary aneurysms who fail medical treatment or those presenting with life-threatening hemoptysis [50].

Xie et al. proposed an algorithm to be used when Behcet disease patients with pulmonary aneurysms require endovascular or surgical management: For patients with peripheral pulmonary artery aneurysms, they recommend a trial of embolizations of affected pulmonary arteries with coils or acrylic glue. If that fails, they recommend surgical interventions such as pulmonary artery ligation, aneurysmorrhaphy, segmentectomy, or lobectomy; For those with aneurysms of the main pulmonary artery, they recommend the use of plugs, occluders, or stent grafts. If that fails, surgical intervention such as pulmonary artery ligation, aneurysmorrhaphy, or pneumonectomy could be a last option. However, pneumonectomy is not encouraged because the pulmonary artery aneurysm may develop on the contralateral side later; For the pulmonary trunk, they recommend a stent graft. If that fails, graft repair or patching will be an option [50].

Coil embolization is a minimally invasive procedure [51]. Intra-saccular coil embolization preserves the pulmonary artery distal to the aneurysm, sparing lung function. However, there is the risk of rupture, migration, or recurrence of hemoptysis [51,52]. When intra-saccular embolization is not feasible or too risky, then embolization proximal and distal to the pulmonary artery aneurysm neck can be performed [51]. In a review of 17 patients with pulmonary artery aneurysms treated with transcatheter coil embolization, immediate control of hemoptysis was achieved in all patients without major complications except for one [52]. However, seven patients (41%) had a recurrence of hemoptysis within five months caused by the development of a bronchosystemic hypervascularization related to the previously occluded pulmonary artery aneurysm [52].

Furthermore, the literature has also reported successful glue embolization of pulmonary artery aneurysms with n-butyl cyanoacrylate (NBCA) [53-55]. NBCA is a commonly used liquid embolic material to treat arteriovenous malformations and can permanently occlude a vessel. It is usually diluted with iodized oil in different ratios depending on the desired polymerization time, adhesiveness, and radiopacity [56].

In addition, vascular plugs can be used in transcatheter embolization of pulmonary artery aneurysms. Vascular plugs are self-expanding cylindrical mesh devices loaded on a delivery catheter and occlude the target site when deployed [57]. The clinical use in the lungs has been mainly in embolizing pulmonary arteriovenous malformations, where it has been used alone or in combination with other techniques, such as embolization coils, with a high success rate [58].

Stent grafts have also been used in pulmonary arteries. They have been reported as effective in managing pulmonary artery pseudoaneurysms while maintaining vascular patency [59,60].

Endovascular treatments of pulmonary artery aneurysms in Behcet disease have fewer risks than surgery. However, patients still have risks similar to other endovascular procedures, including contrast-induced nephropathy, arterial dissection, arterial thrombosis, non-target embolization, and partial or complete lung infarction [51].

Surgical management of pulmonary aneurysm in Behcet disease

Although the risk of surgical procedures is high in pulmonary artery aneurysms, it could be a life-saving procedure in patients with life-threatening hemoptysis [61]. Ideally, surgical treatment should be postponed until active inflammation has subsided, but this is often impossible in emergencies such as life-threatening hemoptysis [62]. The mortality rate in surgically treated patients is high; therefore, surgery should not be an option except for life-threatening situations [63].

The choice of surgical procedure should be determined based on factors such as the aneurysm's size, location, the patient's overall well-being, and the surgeon's experience [63]. In an aneurysmorrhaphy, the surgeon removes the aneurysm and reconstructs the affected pulmonary artery using artificial grafts to replace the damaged artery segment [63-65]. Synthetic grafts are favored over venous grafts due to the lower risk of thrombosis in Behçet's disease patients [63].

Pulmonary artery ligation, though not frequently used in clinical practice, has been reported as successful in pulmonary aneurysms [66]. Pulmonary infarction or necrosis after pulmonary artery ligation is rare because bronchial arteries or collaterals provide an alternative blood supply [66].

Furthermore, successful and unsuccessful cases of segmentectomy, lobectomy, and pneumonectomy have been reported [67-69]. Postoperatively, surgical patients will require close monitoring in an intensive care unit to manage any complications. The patients will require long-term follow-up care to assess the procedure's success and monitor for recurrence. However, the results of surgical therapy for Behçet aneurysms are often disappointing, resulting in graft occlusions, the development of new aneurysms at anastomotic sites, and bleeding from other aneurysms [35,61].

## Conclusions

In conclusion, pulmonary artery aneurysm could lead to hemoptysis, which is the leading cause of death in Behcet disease. However, many of the patients with pulmonary aneurysms have favorable outcomes with immunosuppressive therapy, with corticosteroids and cyclophosphamide being the preferred first-line therapy. However, clinical improvement with infliximab or adalimumab, tumor necrosis factor-alpha (TNFα) blocking agents, have been reported after treatment failure with recommended first-line agents. In patients who fail medical therapy or those with life-threatening hemoptysis, endovascular or surgical intervention is the next option. Endovascular interventions include embolizing affected pulmonary arteries with coils or acrylic glue and using plugs, occluders, or stents. Endovascular interventions usually have fewer adverse effects than surgery. Although the risk of surgical procedures is high in pulmonary artery aneurysms, it could be a life-saving procedure in patients with life-threatening hemoptysis. Surgical options, including pulmonary artery ligation, aneurysmorrhaphy, segmentectomy, lobectomy, or pneumonectomy, could be the last option. However, the results of surgical therapy for Behçet aneurysms are often disappointing.
